# Commentary: Electrophysiological Evidence Reveals Differences between the Recognition of Microexpressions and Macroexpressions

**DOI:** 10.3389/fpsyg.2019.01293

**Published:** 2019-06-11

**Authors:** David Matsumoto, Hyisung C. Hwang

**Affiliations:** ^1^Department of Psychology, San Francisco State University, San Francisco, CA, United States; ^2^Humintell, El Cerrito, CA, United States

**Keywords:** microexpressions, macroexpressions, electrophysioloogy, recognition, EEG

Shen et al. (2016) examined whether perceptions ofshort (40 and 120 ms) and long (200 and 300 ms) expressions were associated with distinctive electrophysiological processes Using an “affective priming paradigm,” the researchers presented pairs of fearful, happy, and neutral expressions with positive and negative emotion words while participants' electroencephalograms (EEG) and Event Related Potentials (ERP) were assessed. Expressions presented at 40 and 120 ms were similar to each other but different from expressions presented at 200 and 300 ms in their ERP and Event Related Spectral Perturbation (ERSP) characteristics. Analyses also suggested that the brain regions responsible for these differences included the inferior temporal gyrus and regions of the frontal lobe and that the left hemisphere was more involved than the right in processing expressions at 200 and 300 ms.

The methods and findings from this study have novel implications concerning facial expressions of emotion (hereafter *FEE*). Below we discuss four such implications with the goal of inspiring further research and insights into this important topic.

## Implications for Emotion Recognition and Processing: Importance of a 200 MS Threshold

Shen et al. (2016) operationalized recognition in their study as a memory task that assessed whether participants remembered seeing face-word pairs. Almost two decades ago we compared different expression presentation speeds and demonstrated that FEE presented at 200 ms were associated with the greatest degree of individual differences in emotion recognition accuracy (Matsumoto et al., 2000). Although our study examined emotion labeling, quite a different task, Shen et al.'s (2016) findings dovetailed nicely with ours in that 200 ms presentations appear to be a threshold at which facial expressions begin to be seen and held in short term memory (corresponding with the Shen et al., 2016, findings) and labeled (corresponding with our findings, Matsumoto et al., 2000). Thus, 200 ms presentation speeds appear to be important for neurophysiological and psychological processes to occur.

## Implications for the Neurophysiology of Brief Facial Emotion Recognition

Shen et al.'s (2016) findings also add to a small but growing literature on the neurophysiological correlates of perceptions of very brief FEE. Although much research has been conducted on the neurophysiology of FEE presented for longer durations (Adolphs, 2002; Atkinson and Adolphs, 2011), the study of very brief FEE is new. In addition to Shen et al. (2016), Zhao et al. (2017) studied neural responses to fearful and surprised expressions presented at 100, 300, or 500 ms and reported both common and separate neural activations associated with their recognition. Relatedly, Peng et al. (2017) developed a dual temporal scale convolutional neural network that could recognize spontaneously produced microexpressions better than feature-based methods. Other novel research involving more traditional physiological assessment (sympathetic responses) combined with observation also suggest promising avenues to deciphering FEE (Wesley et al., 2012).

Shen et al.'s (2016) findings also have implications for other important theoretical questions about expression judgment. For instance, as they suggested, perception of expressions at varying durations have different implications for top-down or bottom-up processing in perceivers. In their study, incongruent word-face combinations produced different electrophysiological profiles than did congruent combinations, implicating top-down processing of the stimuli at even very brief exposures. This suggested that the decoding of emotional stimuli involves the influence of learned rules, labels, values, and associations, providing a cultural component to emotion decoding despite strong biological bases for emotions and expressions in the first place, especially vis-à-vis the type of decoding required.

## Implications for Expression Production

The findings from Shen et al.'s (2016) study and others have implications for expression production. That participants cannot reliably label expressions under 200 ms (Matsumoto et al., 2000) and that the neurophysiological correlates of their perception are different than those of longer expressions (Shen et al., 2016) raise questions about the nature of FEE, and more specifically, the speed of microexpressions. Until recently, the only studies to examine microexpression production had operationalized them as occurring between 1/25th and 1/5th s (i.e., *under* 200 ms) and had shown that they rarely occurred (Porter and ten Brinke, 2008; ten Brinke et al., 2011; Porter et al., 2012; ten Brinke and Porter, 2012). Recently, however, we examined the occurrence of FEE occurring ≤ 0.20, ≤ 0.30, ≤ 0.40, ≤ 0.50, ≤ 1.00, 1.00–6.00, and ≤ 6.00 s in a mock crime paradigm (Matsumoto and Hwang, 2018) and demonstrated that microexpressions ≤ 0.40 and ≤ 0.50 s occurred with sufficient frequency to differentiate truthtellers and liars (We also replicated the previous non-findings that expressions ≤ 0.20 s rarely occurred). These latest findings also dovetailed nicely with Shen et al.'s (2016) and ours (Matsumoto et al., 2000) and collectively have implications concerning which expressions are called micro and which macro. We contend that the term “microexpressions” should be reserved for those that occur faster (i.e., ≤ 0.50 s) than empirically documented speeds of normally occurring, non-suppressed, non-concealed spontaneously produced facial expressions of emotion (0.50–4.00 s; see Ekman et al., 1980, 1998; Ekman and Friesen, 1982; Ekman and Fridlund, 1987; Frank et al., 1993).

Shen et al.'s (2016) findings also have implications for other important theoretical questions about expression production. For instance, expressions at different durations have different implications for a voluntary-involuntarily produced distinction, as well as conceptual issues concerning possible overlap with macroexpressions; future research needs to examine these issues. Other questions exist: Is there a duration criterion that can differentiate when an expression is voluntarily as opposed to involuntarily produced? Is there a duration criterion concerning when expressions are signs of concealed emotional states? Are there morphological differences in expressions—micro and macro—produced with these different psychological states? And are there different neural correlates of these different psychological processes? All of these are questions that the Shen et al. (2016) article implies and that should be examined in the future.

## Implications for Deception Detection

Finally, the findings by Shen et al. (2016) and a growing number of other studies have implications for deception detection. As mentioned above, microexpressions do occur when individuals lie and they differentiate truthtellers from liars (Matsumoto and Hwang, 2018). Microexpression recognition ability is associated with deception detection accuracy (Frank and Ekman, 1997; Matsumoto et al., 2014) and microexpression recognition skills can improve through training and are retained after training (Matsumoto and Hwang, 2011; Hurley, 2012). Individual difference variables are associated with microexpression recognition ability, including openness, conscientiousness, affective empathy, and emotion dysregulation (Matsumoto et al., 2000; Hurley, 2012; Hurley et al., 2014; Svetieva and Frank, 2016). And brain activity differs when individuals perceive microexpressions above a 200 ms threshold (Shen et al., 2016). For all these reasons, microexpression recognition may be one (of many) important skills in detecting deception, having implications for a wide range of real life concerns as well as for future research and theory.

## Author Contributions

DM and HH authors contributed equally to the ideas presented. The DM wrote the first draft of the paper. Both authors contributed equally to editing the first draft to its final version.

### Conflict of Interest Statement

The authors declare that they are employees of Humintell, a for-profit company that sells microexpression related products.

## References

[B1] Adolphs R. (2002). Recognizing emotion from facial expressions: psychological and neurological mechanisms. Behav. Cogn. Neurosci. Rev. 1, 21–62. 10.1177/1534582302001001003 17715585

[B2] Atkinson A. P. Adolphs R. (2011). The neuropsychology of face perception: beyond simple dissociations and functional selectivity. Philos. Trans. R. Soc. B 366, 1726–1738. 10.1098/rstb.2010.0349 21536556 PMC3130374

[B3] Ekman P. Fridlund A. J. (1987). Assessment of facial behavior in affective disorders, in Depression and Expressive Behavior, ed Maser J. D. (Hillsdale, NK: Lawrence Erlbaum Associates), 37–56.

[B4] Ekman P. Friesen W. V. (1982). Felt, false, and miserable smiles. J. Nonverb. Behav. 6, 238–258. 10.1007/BF00987191

[B5] Ekman P. Friesen W. V. Ancoli S. (1980). Facial signs of emotional experience. J. Pers. Soc. Psychol. 39, 1125–1134. 10.1037/h0077722

[B6] Ekman P. Matsumoto D. Friesen W. V. (1998). Facial expressions in affective disorders, in What the Face Reveals: Basic and Applied Studied of Spontaneous Expression Using the Facial Action Coding System (FACS), eds Ekman P. Rosenberg E. (New York, NY: Oxford University Press), 331–341.

[B7] Frank M. G. Ekman P. (1997). The ability to detect deceit generalizes across different types of high-stake lies. J. Pers. Soc. Psychol. 72, 1429–1439. 10.1037/0022-3514.72.6.1429 9177024

[B8] Frank M. G. Ekman P. Friesen W. V. (1993). Behavioral markers and recognizability of the smile of enjoyment. J. Pers. Soc. Psychol. 64, 83–93. 10.1037/0022-3514.64.1.83 8421253

[B9] Hurley C. M. (2012). Do you see what I see? Learning to detect micro expressions of emotion. Motiv. Emot. 36, 371–381. 10.1007/s11031-011-9257-2

[B10] Hurley C. M. Anker A. E. Frank M. G. Matsumoto D. Hwang H. C. (2014). Background factors predicting accuracy and improvement in micro expression recognition. Motiv. Emot. 38, 700–714. 10.1007/s11031-014-9410-9

[B11] Matsumoto D. Hwang H. C. (2011). Evidence for training the ability to read microexpressions of emotion. Motiv. Emot. 35, 181–191. 10.1007/s11031-011-9212-2

[B12] Matsumoto D. Hwang H. C. (2018). Microexpressions differentiate truths from lies about future malicious intent. Front. Psychol. 9:2545. 10.3389/fpsyg.2018.02545 30618966 PMC6305322

[B13] Matsumoto D. Hwang H. C. Skinner L. G. Frank M. G. (2014). Positive effects in detecting lies from training to recognize behavioral anomalies. J. Police Crim. Psychol. 29, 28–35. 10.1007/s11896-012-9115-5

[B14] Matsumoto D. LeRoux J. A. Wilson-Cohn C. Raroque J. Kooken K. Ekman P. (2000). A new test tomeasure emotion recognition ability: Matsumoto and Ekman's Japanese and Caucasian Brief Affect Recognition Test (JACBART). J. Nonverb. Behav. 24, 179–209. 10.1023/A:1006668120583

[B15] Peng M. Wang C. Chen T. Liu G. Fu X. (2017). Dual temporal scale convolutional neural network for micro-expression recognition. Front. Psychol. 8:1745. 10.3389/fpsyg.2017.01745 29081753 PMC5645805

[B16] Porter S. ten Brinke L. (2008). Reading between the lies: Identifying concealed and falsified emotions in universal facial expressions. Psychol. Sci. 19, 508–514. 10.1111/j.1467-9280.2008.02116.x 18466413

[B17] Porter S. ten Brinke L. Wallace B. (2012). Secrets and lies: involuntary leakage in deceptive facial expressions as a function of emotional intensity. J. Nonverb. Behav. 36, 23–37. 10.1007/s10919-011-0120-7

[B18] Shen X. Wu Q. Zhao K. Fu X. (2016). Electrophysiological evidence reveals differences between the recognition of microexpressions and macroexpressions. Front. Psychol. 7:1346. 10.3389/fpsyg.2016.01346 27630610 PMC5005928

[B19] Svetieva E. Frank M. G. (2016). Empathy, emotion dysregulation, and enhanced microexpression recognition ability. Motiv. Emot. 40, 309–320. 10.1007/s11031-015-9528-4

[B20] ten Brinke L. MacDonald S. Porter S. O'Connor B. (2011). Crocodile tears: facial, verbal and body language behaviours associated with genuine and fabricated remorse. Law Hum. Behav. 10.1007/s10979-011-9265-5 22471385

[B21] ten Brinke L. Porter S. (2012). Cry me a river: identifying the behavioral consequences of extremely high-stakes interpersonal deception. Law Hum. Behav. 36, 469–477. 10.1037/h0093929 23205594

[B22] Wesley A. Lindner P. Pavlidis I. (2012). Eustressed or distressed?: combining physiology with observation in user studies, in Paper presented at the CHI '12 Extended Abstracts on Human Factors in Computing Systems (Austin, TX). 10.1145/2212776.2212811

[B23] Zhao K. Zhao J. Zhang M. Cui Q. Fu X. (2017). Neural responses to rapid facial expressions of fear and surprise. Front. Psychol. 8:761. 10.3389/fpsyg.2017.00761 28539909 PMC5424260

